# On the reduction in the effects of radiation damage to two-dimensional crystals of organic and biological molecules at liquid-helium temperature

**DOI:** 10.1016/j.ultramic.2022.113512

**Published:** 2022-07

**Authors:** Katerina Naydenova, Akiko Kamegawa, Mathew J. Peet, Richard Henderson, Yoshinori Fujiyoshi, Christopher J. Russo

**Affiliations:** a MRC Laboratory of Molecular Biology, Francis Crick Avenue, Cambridge CB2 0QH, UK; b Cellular and Structural Physiology Laboratory (CeSPL), Tokyo Medical and Dental University, Yushima, Bunkyo-ku, Tokyo, Japan

**Keywords:** Radiation damage, cryoEM, 2D crystallography, Liquid helium, Cryomicroscopy

## Abstract

We have studied the fading of electron diffraction spots from two-dimensional (2D) crystals of paraffin (C_44_H_90_), purple membrane (bacteriorhodopsin) and aquaporin 4 (AQP4) at stage temperatures between 4K and 100K. We observed that the diffraction spots at resolutions between 3 Å and 20 Å fade more slowly at liquid-helium temperatures compared to liquid-nitrogen temperatures, by a factor of between 1.2 and 1.8, depending on the specimens. If the reduction in the effective rate of radiation damage for 2D crystals at liquid-helium temperature (as measured by spot fading) can be shown to extend to macromolecular assemblies embedded in amorphous ice, this would suggest that valuable improvements to electron cryomicroscopy (cryoEM) of biological specimens could be made by reducing the temperature of the specimens under irradiation below what is obtainable using standard liquid-nitrogen cryostats.

## Highlights

• The fading of electron diffraction spots from 2D crystals is measured at 4–100K.
• Specimens studied are paraffin, purple membrane and aquaporin 4.
• Spots fade 1.2–1.8× slower at liquid helium vs. nitrogen.
• CryoEM might be improved at temperatures below those of liquid nitrogen.

## 1 Introduction

In the last few years there has been a surge in the use of single-particle electron cryomicroscopy for the determination of the high-resolution structures of macromolecular assemblies [1]. Virtually all these structure determinations used specimens embedded in amorphous ice prepared with Dubochet’s plunge-freeze method, and imaged at temperatures near that of liquid nitrogen (77–100K) [2]. At these temperatures, the diffusion constant of water (in the absence of electron irradiation) is vanishingly small [3], which affords the preservation of molecular structure indefinitely by the complete immobilisation of the water molecules. In addition, keeping the specimen at low temperature during imaging with high energy particles reduces any effects downstream of primary damage under irradiation. Primary damage, at the event of the interaction of the high-energy electron with the specimen, is a temperature-independent process, far from thermodynamic equilibrium, but it is followed by thermally activated energy transfer and diffusion processes. The details of how radiation damage from these is reduced at low temperatures remain of interest; in particular, we wish to begin reexamining the question, what is the optimum temperature for cryoEM of biological specimens?

After many investigations by several groups, it was generally agreed that there was an important, approximately 4-fold advantage in using liquid-nitrogen temperature compared with room temperature for structure determination of biological specimens in the electron microscope [4]. This observation is supported by work on radiation damage mechanisms in crystals at room temperature: interaction with high-energy electrons causes ionisation or radical formation in the specimen, which is a temperature-independent phenomenon, but the rate of diffusion to nearby areas of the specimen, depends strongly on temperature [5]. The diffusion rate of radiolytic products away from their initial location, and thus their loss, is reduced with temperature, which slows the consequences of bond breakage on the integrity of the overall structure. While it was found that cryogenic cooling was beneficial, there was less agreement about a further reduction in radiation damage at lower temperatures closer to absolute zero [4]. Two decades later, when it became possible to make a direct comparison between images of specimens at liquid-nitrogen and liquid-helium temperatures under otherwise identical imaging conditions [6], it became clear that the problem of beam-induced specimen motion was more severe at liquid-helium temperature than at liquid-nitrogen temperature. At that time, it was found that for recording images either for single-particle cryoEM [6] or electron cryotomography [7], liquid-helium temperature was actually worse than liquid-nitrogen temperature. A recent comparison of single-particle images of apoferritin [8] which used all-gold grids that reduce movement of the specimen [9], showed little difference in resolution and twofold greater beam-induced motion at liquid-helium temperature, even with these all-gold supports. Now, following the success of single-particle cryoEM at liquid-nitrogen temperatures, it seems timely to make a more careful comparison of the effects of radiation damage at temperatures at and below that of liquid nitrogen. We hope this might point the way to further improvements in single-particle cryoEM technology, and resolve some of the discrepancies in the literature on the subject.

There has been a long history of publications reporting studies of the consequences of radiation damage to organic and biological molecules by observing the fading of diffraction spots from crystals. Early electron diffraction work [10] at room temperature showed quantitative rate differences between the loss of order in thin valine and adenosine crystals by electron diffraction, with adenosine being about 8-fold more stable than valine. In 1979, Hayward and Glaeser showed more than a four-fold increase in the lifetime of purple membrane diffraction by cooling to around −120°C [11]. The early excitement that much greater improvements could be obtained by use of liquid-helium cooling to reach the 4–20K temperature range [12], [13] evolved into the formation of an International Experimental Study Group [4], which studied spot fading in 2D crystals of n-paraffin (C_44_H_90_) and purple membrane. The consensus conclusion was that “specimens last approximately 3 to 5 times as long when the specimen is cooled to about 150K” compared with room temperature. However, they also concluded that “we have been unable to find definite evidence that there is an improvement in radiation resistance on going from liquid-nitrogen to liquid-helium cooling”. A later review [14] included plots of the fading at 5.5 Å for catalase diffraction and at 9 Å for tRNA diffraction. These results confirmed the generally accepted factor of ∼4-fold improvement at around 70K, and also suggested a further increase to 10-fold at <20K and 20-fold at <8 K, but the author of the review has less confidence in the quantitative values because there was insufficient data for accurate measurements of cryo-protection factors. Previously published electron radiation damage measurements on a wide variety of different specimens, at different temperatures and electron energies, are summarised in Supplementary Table A1 [15], [16], [17], [18], [19], [20].

The progressive reduction in data quality with increased electron dose is usually described using a Gaussian approximation akin to the Debye–Waller equation for thermal vibration of atoms in a lattice. Using this approximation, the decay in the intensity I of the diffraction spots, or the Fourier intensities in an image, can be characterised by their fading with dose according to

(1) $$I=I_{0}e^{-B\left(f\right)k^{2}/2},$$

where B is the Debye–Waller factor, k is the spatial frequency, f is the fluence in electrons per unit area and $I_{0}$ is the intensity of the spot in the undamaged structure. As radiation damage destroys the features of the molecules, the magnitude of B increases and the intensity decays with fluence/dose. This decay with dose is well-established in data obtained from electron diffraction from 2D crystals [10] and from single-particle cryoEM images [21], [22], [23]. Spot fading at a particular resolution is described by a characteristic electron fluence, $N_{e}$ or lifetime, for fading from an initial average intensity of $I_{0}$ to an intensity I, where $I/I_{0}=1/e$. Thus, the change in B per unit fluence can be related to $N_{e}$ by

(2) $$\Delta B=2d^{2}/N_{e},$$

where d=1/k is the resolution for which $N_{e}$ is measured. The typical loss of information in the earliest part of the exposure in images, during the first few e${}^{-}$/Å${}^{2}$, is an exception from this theory, as movement of the specimen causes blurring of the image not related to radiation damage. An analysis of the various factors that degrade single-particle cryoEM images is beyond the scope of this paper but is briefly summarised in [24]. Additionally, excellent discussions of radiation damage in many solid state materials more widely in TEM and SEM can be found in the work of Egerton [25].

Alongside previous work on radiation damage using electron diffraction, there has also been a considerable body of work exploring radiation damage by X-ray diffraction of three-dimensional (3D) crystals of proteins and other macromolecular assemblies [26], [27], [28]. Meents et al. [28] showed that hydrogen gas is the main product of radiation damage to insulin or elastase crystals, with smaller quantities of methane, carbon monoxide and carbon dioxide also being produced. It is believed that the underlying mechanism of radiation damage is essentially the same whether the ionisation is triggered by primary or secondary electrons in cryoEM or by photoelectrons produced by X-ray absorption [29], [30]. A rigorous analysis of the resolution and dose dependence of X-ray damage in protein crystals, examining a wide range of published data, is given in a recent paper [31]. The physics underlying basic experimental trends in radiation damage at 100K can be well-described in terms of a random, local blurring of the structure, characterised by a B(f) that increases linearly with dose [32]. This rate of global radiation damage was shown to be independent of the dose rate once the crystal is cryogenically cooled [33]. There has been less work using X-ray diffraction at temperatures lower than that of liquid nitrogen. For two different specimens the reported improvement upon cooling with liquid helium was between 25% and 40% relative to liquid nitrogen [34], [35], [36], [37]. At least one investigation has shown a greater than 8-fold decrease in the rate of radiation damage at 40K compared to 110K for metal centres in metalloproteins, which are especially sensitive to reduction due to radiation damage [38].

The specific purpose of this work is to make a more accurate comparison of radiation damage at temperatures between 4K and 100K by measuring spot fading in electron diffraction patterns using two different microscopes that could be operated interchangeably at liquid-helium and liquid-nitrogen temperatures. Here, we again use paraffin and purple membrane as test specimens but also include 2D crystals of aquaporin (AQP4). By using these same three specimens under identical illumination conditions in the same microscopes, and with the same detectors and the same analysis, we now show that there is indeed a useful reduction in the rate of diffraction spot fading at temperatures lower than that of liquid nitrogen. If this reduction in radiation damage can be translated to imaging vitreous specimens in cryoEM, it would have wide ranging implications: the amount of information obtainable from a given specimen before the onset of radiation damage is increased, and thus smaller particles can be detected and imaged in a specimen of a given thickness [39].

## 2 Materials and methods

### 2.1 Specimen preparation

Since the objective of this work was to measure rigorously the relative rates of spot fading and to demonstrate reproducibility between microscopes and institutions, specimens were prepared independently in two countries — in the UK (MRC Laboratory of Molecular Biology, Cambridge) and at two locations in Japan (1. Cellular and Structural Physiology Institute at Nagoya University, Nagoya, 2. Cellular and Structural Physiology Laboratory at Tokyo Medical and Dental University, Tokyo).

In Cambridge, specimens of C_44_H_90_ paraffin were made by applying 2μl of a nearly saturated solution of C_44_H_90_ in hexane (Supelco Inc.) to a film of amorphous carbon that had previously been floated off mica on to 400 mesh copper EM grids, and allowed to dry in air. Crystals of C_44_H_90_ paraffin one molecule thick formed as described in [40]. Also, in Cambridge, specimens of purple membrane were prepared as described in [41]. Briefly, 400 mesh grids were coated with amorphous carbon, that had been floated off mica. These grids were pre-treated with a 4μl drop of 1% ovalbumin (Sigma), then washed with 2 drops of water, then dried. This ovalbumin pre-treatment prevents direct contact between the purple membrane and the carbon film, which often introduces disorder. A 2μl drop of fused purple membranes (1 mg/ml) was then applied to the pre-treated grids, blotted and then, while the grids were still wet, a final 2μl drop of 1% glucose was applied, blotted and dried in air. The glucose creates a hydrophilic environment that preserves the crystalline order. Grids were then immersed in liquid nitrogen where they could be stored indefinitely. In Cambridge, the same procedure was used to prepare grids of aquaporin AQP4, sent from Nagoya/Tokyo.

In Nagoya/Tokyo, the method of specimen preparation for purple membrane and aquaporin was slightly different. Briefly, a film of amorphous carbon was evaporated on freshly cleaved mica, using a sufficiently low current to prevent any sparking. These conditions gave smooth and stable carbon films. The mica sheets were cut into roughly 4 mm square pieces, and the film was floated off onto distilled water and picked up with a molybdenum grid. The distilled water was exchanged with crystallisation buffer (10 mM MES pH 6.0, 100 mM NaCl, 50 mM MgCl${}_{2}$, 2 mM DTT, 1% v/v gycerol) including trehalose at a final concentration of 7% (w/v) by successively touching the solution to 3 fresh droplets of buffer that had been placed on a piece of parafilm. The grid was then turned over, and 2 μL of the sample solution including AQP4 crystals [42] or purple membranes was applied by pipetting several times. The grid was again turned over and put on a piece of filter paper by which excess sample volume was removed from the opposite side of the carbon. Finally, the grid was quickly plunged into liquid nitrogen using a KF80 plunge-freezer (Reichert-Leica). Some AQP4 grids prepared in Nagoya/Tokyo were also shipped at liquid-nitrogen temperature to Cambridge where electron diffraction was performed, to control for the possible influence of the slightly different sample preparation procedures.

### 2.2 Recording electron diffraction dose series

In Cambridge, electron diffraction patterns were recorded on a FEI Polara G2 microscope with the specimens cooled using either liquid nitrogen or liquid helium in the inner Dewar. The stage temperature, as measured using the sensor nearest to the specimen, was 8 K when using liquid nitrogen, and 12–15K when using liquid helium. The beam current was calibrated using a Faraday cup and picoammeter, and the diameter of the illuminating beam measured using the microscope in imaging mode with magnification calibration using a cross-grating grid. Crystals were also observed at low magnification by defocusing the central spot of the diffraction pattern, before and after each exposure series to verify that the crystal and beam did not drift during irradiation. A nominal camera length of 930 mm was used with the energy set to 300 keV. Diffraction patterns were acquired on a Gatan Orius SC200B camera with 2048 × 2048 pixels. The screen current was monitored throughout each experiment, and on different occasions had specific values between 82 and 181 pA, measured without a specimen in the beam path. The typical variation in screen current in the duration of each experiment was <1%. Beam diameters from 1.6 to 3.4μm were used, and only crystals with dimensions larger than the beam were selected (typically 3 to 5 μm). The electron flux was calculated from these two measurements, and was multiplied by the total exposure time to find the cumulative fluence at each measurement point. For C_44_H_90_, a series of 40 × 0.05 s exposures was acquired with the diffraction patterns slightly defocused to avoid saturating the detector. The 0.05 s exposures were separated by 1.0 s irradiation, at an electron flux of 1.1 e${}^{-}$/Å${}^{2}$/s. For purple membrane and aquaporin, a series of 8–16 contiguous 0.7–1.5 s exposures at measured fluxes of 2.5–3.5 e${}^{-}$/Å${}^{2}$/s was acquired for each crystal.

In Nagoya, electron diffraction patterns were recorded on a JEM-3000SFF (JEOL) electron microscope equipped with a liquid-helium stage [14], and operated at 300 kV. The cryostat temperature was maintained at approximately 100K when using liquid nitrogen, and a nominal 4K when using liquid helium. Crystals were observed at low magnification as above. The nominal camera length was 800 mm and the diffraction patterns were recorded on a Gatan Orius SC200 camera with 2048 × 2048 pixels. A series of exposures was recorded at electron fluxes of 0.073–0.078 e${}^{-}$/Å${}^{2}$/s, where each diffraction pattern was acquired during a 10 or 15 second exposure, followed by a 60 or 65 second interval of irradiation before the next pattern. The beam diameter was about 11 μm in the specimen plane. The electron fluxes were calculated by counting on a Gatan K2 Summit camera.

Each pair of liquid helium/nitrogen measurements was done with crystals on the same grid using the same microscope and the same detector, during the same data collection session with constant electron irradiation conditions for the session. Only the temperature was changed between measurements.

### 2.3 Computer processing of diffraction series, fading and cell dimension

For C_44_H_90_ paraffin, the centres of the 5 or 6 individual spots with indices (1,1), (1,−1) and (2,0) and their corresponding Friedel mates were used to integrate the background-corrected diffracted intensity for each spot on each frame as the diffraction spots faded, using the display program Ximdisp [43] manually, as described in [44]. The intensity measurements shown in Fig. 2 were made on every second or third pattern in the exposure series. Electron diffraction patterns from purple membrane or aquaporin recorded in either laboratory were initially processed as described previously [41], [45], [46]. Briefly, the MRC programs BACKAUTO, AUTOINDEX and PICKAUTO [47] were used, in which the diffraction pattern centre, radial background and lattice parameters were automatically indexed and refined on the first diffraction pattern in each series, and then carried over to provide the starting parameters in the subsequent (weaker) exposures in the series. Then the average intensities in three resolution zones, centred at 9 Å, 5 Å and 4 Å, were averaged for 2 to 5 crystals for each experiment at each temperature, and plotted as a function of electron fluence as a ratio to the average intensity of the same zone in the first frame, as described in [44]. The cell dimensions for each crystal as a function of cumulative fluence were refined in PICKAUTO as an intrinsic part of this procedure.

## 3 Results

**Fig. 1:**
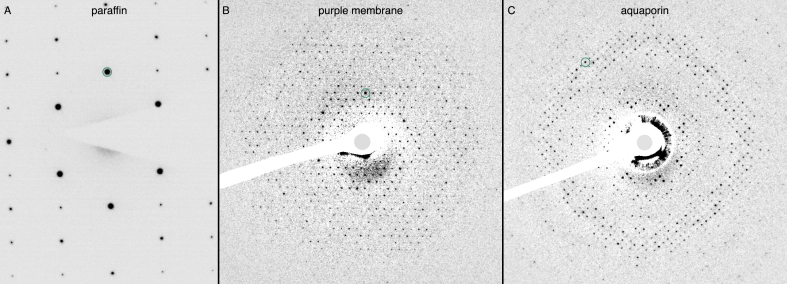
**Diffraction patterns from 2D crystals used for radiation damage measurements.** The diffraction patterns from **(A)** paraffin, **(B)** purple membrane, and **(C)** aquaporin AQP4 crystals are shown, recorded at 84K with 1.1 e${}^{-}$/Å${}^{2}$ at 300 kV accelerating voltage. The contrast has been inverted in these and the background has been subtracted in panels **(B)** and **(C)**, as described in the Methods. The following reflections are circled for scale: (2, 0) for paraffin **(A)**, (4, 3) for purple membrane **(B)**, and (17, 1) for aquaporin **(C)**.

**Fig. 2:**
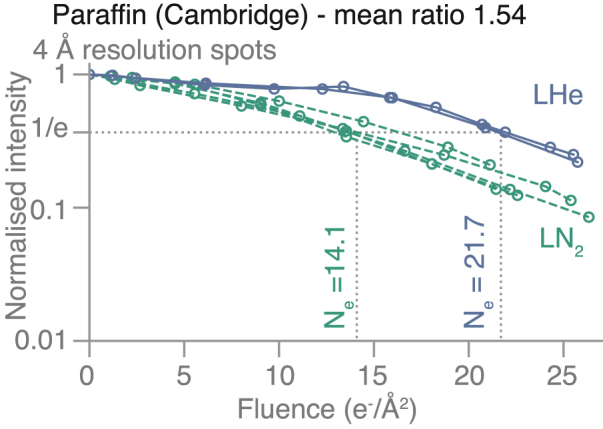
**Measurement of spot fading on Paraffin crystals at liquid-nitrogen(LN**${}_{2}$, ***dashed green line*) and liquid-helium temperature (LHe, *solid blue line*).** The normalised intensity of the diffraction spots within the 4 Å resolution band is plotted on a logarithmic scale versus to cumulative electron fluence. The fluence at which the averaged intensity reaches 1/e of its initial value is labelled as $N_{e}$ for the two temperatures. The ratio $N_{e}$(LHe)/$N_{e}$(LN${}_{2}$) was found to be 1.54.

Examples of diffraction patterns from the first exposure of the three different specimens, before significant radiation damage had occurred, are shown in Fig. 1. The initial diffraction patterns from all three specimens extend well beyond 3 Å, but we have restricted the analysis of spot fading to around 3.5 Å resolution for these measurements. This allows for accurate spot fading measurements throughout at least 20 e${}^{-}$/Å${}^{2}$ of exposure. The spot fading for the three diffraction spots at 4 Å resolution for C_44_H_90_ paraffin is shown in Fig. 2, with a 1.5-fold increased lifetime at 13K compared with 84K (Table 1). The cell dimensions and space group for the paraffin crystals are a=7.4, b=5.0, with space group P12${}_{1}$, with the (1,0) reflection in a systematic absence, so the first three spots are at 4.1, 4.1, and 3.7 Å resolution, and these have been averaged to produce the data shown in Fig. 2. Paraffin is the only one out of the three specimens used in this work, for which we observed a latent dose effect: the diffraction spots do not start fading immediately upon irradiation. We attribute this to deformations in the crystal at the onset of irradiation [44]. This effect was the reason we focused the investigations on other crystals (purple membrane and aquaporin) for the purpose of this study.

**Fig. 3:**
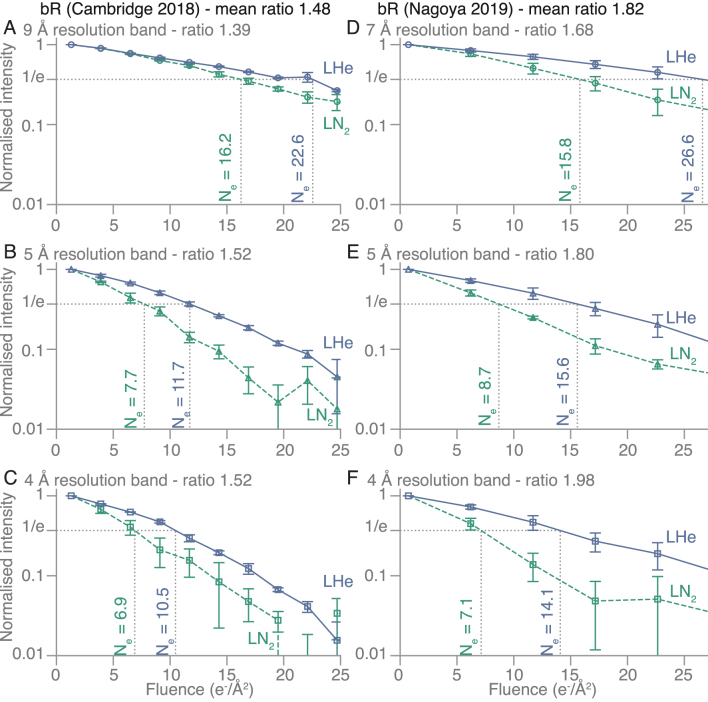
**Measurements of spot fading on purple membrane crystals at liquid-nitrogen(LN**${}_{2}$, ***dashed green line*) and liquid-helium (LHe, *solid blue line*) temperature from two independent experiments.** The fading of diffraction spots in three resolution bands was analysed in series of diffraction patterns from purple membrane crystals recorded on two different electron microscopes: in Cambridge **(A–C)** and Nagoya **(D–F)**. The normalised intensity of the diffraction spots within each resolution band is plotted on a logarithmic scale versus to cumulative electron fluence. The fluence at which the intensity reaches 1/e of its initial value is labelled as $N_{e}$ for each fading curve. The ratios $N_{e}$(LHe)/$N_{e}$(LN${}_{2}$) are shown for each panel. The markers show mean values from all averaged patterns, and the error bars show standard deviations. For **(A–C)**n=2 selected diffraction pattern series were averaged for each of the temperatures, and for **(D–F)**n=4.

**Fig. 4:**
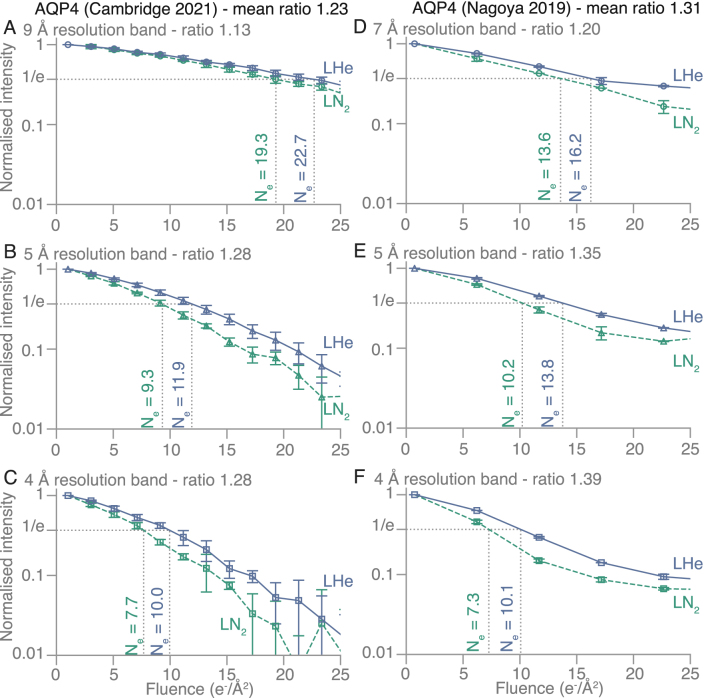
**Measurements of spot fading on aquaporin crystals at liquid-nitrogen(LN**${}_{2}$, ***dashed green line*) and liquid-helium (LHe, *solid blue line*) temperature from two independent experiments.** The fading of diffraction spots in three resolution bands was analysed in series of diffraction patterns from aquaporin crystals recorded on two different microscopes: in Cambridge for **(A–C)** and in Nagoya for **(D–F)**. The normalised intensity of the diffraction spots within each resolution band is plotted on a logarithmic scale versus to cumulative electron fluence. The fluence at which the intensity reaches 1/e of its initial value is labelled as $N_{e}$ for each fading curve. The ratios $N_{e}$(LHe)/$N_{e}$(LN${}_{2}$) are shown for each panel. The markers show mean values from all averaged patterns, and the error bars show standard deviations. For **(A–C)**n=4 selected diffraction pattern series were averaged for the liquid-nitrogen condition, and n=5 - for the liquid helium, and for **(D–F)**n=2 for each temperature.

Since the cell dimensions for purple membrane (a=63 Å) and aquaporin (a=69 Å) are about 10× larger than for paraffin, the 2D protein crystals have more than 100× the number of diffraction spots out to 3.5 Å resolution. For purple membrane, there are about 750 diffraction spots out to 3.5 Å, consisting of over 300 Friedel pairs. The diffraction spots fade faster at high resolution, but many of the highest resolution spots are relatively faint, so we have combined the measurements into three resolution zones to show the fading in Fig. 3 and Supplementary Fig. A1. To minimise the influence of any flux calibration errors, we only make comparisons between data acquired at the two temperatures on the same microscope during the same experiment. In this way, we find an increase in lifetime for the spots in all resolution shells by a factor of 1.4 to 2.0 with liquid-helium cooling compared to liquid nitrogen. The values of $N_{e}$ and corresponding increase in B, with exposure are also shown for purple membrane in Table 2. The characteristic dose is lower at higher resolution since high-resolution spots fade faster. For aquaporin, there are slightly more spots than for purple membrane, but we have used the same procedure and the same three resolution zones to quantitate the spot fading. This is shown in Fig. 4, Supplementary Fig. A2 and Table 3. Similarly, the lifetime of spots in all three resolution shells is increased by a factor of 1.1 to 1.4 with liquid-helium cooling compared to liquid nitrogen. For both purple membrane and aquaporin, the improvement afforded by liquid-helium cooling is slightly larger for the higher-resolution bands (4–5 Å) than for the low-resolution ones (7–9 Å).

Table 4 shows that there is an increase in the cell dimensions of all three 2D crystal specimens during irradiation, which parallels the spot fading and increased disorder. This increase progresses throughout the electron exposure, until the diffraction spots disappear or become few enough that the error in the determination of cell dimensions is greater than the expected change in magnitude. For the paraffin crystals, we find a bigger increase in cell dimension at liquid-helium temperature than at liquid-nitrogen temperature ( Table 4).

**Table 1:** Summary of fading lifetimes $N_{e}$ and ΔB for paraffin diffraction patterns.

Quantity	80K	13K	$N_{e}$(80K)$/N_{e}$(13K)
$N_{e}$ (4 Å)	14.1	21.7	1.5

ΔB (Å ${}^{2}$/(e${}^{-}$/Å ${}^{2}$))	2.3	1.5	1.5

**Table 2:** Summary of fading lifetimes $N_{e}$ and ΔB for purple membrane diffraction patterns.

Quantity	80K	13K	4K	$N_{e}$(80K)$/N_{e}$(13K)	$N_{e}$(80K)$/N_{e}$(4K)
$N_{e}$ (9 Å)	16.2	22.6		1.4	
	15.8		26.6		1.7
$N_{e}$ (5 Å)	7.7	11.7		1.5	
	8.7		15.6		1.8
$N_{e}$ (4 Å)	6.9	10.5		1.5	
	7.1		14.1		2.0

ΔB (Å ${}^{2}$/(e${}^{-}$/Å ${}^{2}$))	5–6	3–4	2–3	1.5	1.9

**Table 3:** Summary of fading lifetimes $N_{e}$ and ΔB for aquaporin diffraction patterns.

Quantity	80K	13K	4K	$N_{e}$(80K)$/N_{e}$(13K)	$N_{e}$(80K)$/N_{e}$(4K)
$N_{e}$ (9 Å)	19.3	22.7		1.1	
	13.6		16.2		1.2
$N_{e}$ (5 Å)	9.3	11.9		1.3	
	10.2		13.8		1.4
$N_{e}$ (4 Å)	7.7	10.0		1.3	
	7.3		10.1		1.4

ΔB (Å ${}^{2}$/(e${}^{-}$/Å ${}^{2}$))	4–5	3–4	3–4	1.3	1.4

**Table 4:** Cell dimension expansion during irradiation with the specified electron fluence.

2D crystal	Cell	Fluence	Expansion			Expansion
		(e${}^{-}$/Å ${}^{2}$)	4K	13K	100K	(Å)
Paraffin	a= 7.4 Å, b= 5.0 Å	50	–	0.86 ± 0.1%	0.45 ± 0.2%	≈ 0.04
	(P12${}_{1}$)					
Purple	a= 62.5 Å	25	0.4 ± 0.1%	0.45 ± 0.2%	0.45 ± 0.1%	≈ 0.25
membrane	(P3)					
Aquaporin	a= 69.0 Å	25	0.15 ± 0.15%	–	0.1 ± 0.1%	≈ 0.12
AQP4	(P42${}_{1}$2)					

## 4 Discussion

### 4.1 Paraffin, purple membrane, aquaporin: reduced damage using liquid helium

We demonstrate a significant and reliable increase in the lifetime of diffraction spots from 2D crystals of organic or biological specimens, as their temperature is lowered from near liquid-nitrogen temperature to near liquid-helium temperature. This increase was reproduced in two different laboratories using two different electron cryomicroscopes, three specimens, and different specimen preparation methods. The measurements carried out in Cambridge (Supplementary Fig. A1A-C, A2A-C,G-I) show a narrower distribution (ranging from 1.1 to 1.5) of the improvement in lifetime with the Polara liquid-helium cryostat reaching a temperature of 12 to 15K. The measurements carried out in Nagoya show a wider distribution of improved lifetimes, from 1.1 up to 2.0 (Supplementary Fig. A1D-L, A2D-F,J-L). Since each liquid helium/nitrogen comparison is done with crystals on the same grid using the same microscope and the same detector, one explanation for the broader spread in radiation protection factors measured on the microscope in Nagoya is variance in specimen temperatures. The temperature of the specimen, reached with liquid-helium cooling, is not necessarily equal to that of the microscope stage (4K in Nagoya and 12–15K in Cambridge), due to the low thermal conductivity of most materials in this range. It is possible that for the microscope in Nagoya, the specimen temperature varied over a wider range (from 8 to 20K) and included temperatures lower than the 12–15K in Cambridge. The temperature range of 8K to 20K was estimated from previous observations of diffraction patterns from solid Ne and N${}_{2}$ condensed on the specimen supports *in situ*, respectively. Temperature measurements of electron beam illuminated specimen areas are difficult, especially for small specimen regions in thin specimens. Therefore, attempts were made to confirm specimen temperatures (not stage temperatures) using a gas introduction system [14], [48]. Although many attempts were made to observe the diffraction pattern of solid H${}_{2}$, which should be observable below 3K, no H${}_{2}$ ring pattern was ever observed even using superfluid helium which cooled down the stage to a temperature of 1.5K, presumably because illumination by the electron beam increased the local temperature of the specimen to higher than 3K. When Ne and N${}_{2}$ rings in diffraction patterns were observed, this indicated specimen had temperatures of 8K and 20K, respectively, but depended on beam illumination conditions as well as specimen conditions. In practice, keeping the temperature below 8K was difficult because the observed Ne ring was extremely weak, possibly because the specimen in the electron beam illuminated area could easily reach a higher temperature than that of Ne sublimation [14]. These results leave open the possibility that the increased lifetime of diffraction may be even greater than the average of the measurements we report here, if a temperature of 1–5K could be reliably obtained in a cryogenic electron microscope. We also conclude that having an *in situ* form of temperature measurement incorporated directly in the specimen or perhaps the specimen support, that was accurate in range of temperatures from 1–100K, would be highly desirable.

### 4.2 Mechanism of radiation damage at cryogenic temperatures

We suggest that the increased lifetime as the temperature is lowered is likely to be due to the reduced mobility of the small molecular weight radiation products (H${}^{•}$, O${}^{•}$, HO${}^{•}$, H${}_{2}$, CO, O${}_{2}$, N${}_{2}$, CH${}_{4}$, C${}_{2}$H${}_{6}$, etc.). Some of these fragments are gases at liquid-nitrogen temperature that can diffuse out of the specimen into the microscope vacuum, leaving cavities in the structure which may collapse and create disorder more rapidly than at liquid-helium temperature. Some may also be solids with high vapour pressure meaning they sublime away once they reach the surface of the thin specimen. At liquid-helium temperature, the structures are still damaged by the electron beam, but the irradiated structures kept at this lower temperature bear a closer resemblance to the undamaged structures than the structures after irradiation with the same dose at liquid-nitrogen temperature. The measurements of increased diffraction lifetime upon cooling in this work reflect the rates of global radiation damage, averaged over all atoms in the crystals. At especially radiation-sensitive sites, such as metal ions, disulphide bridges, and carboxyl groups, the improvement due the liquid-helium cooling may be greater than the average value of 1.2–1.8× measured here. We also observe that the cell dimensions increase during irradiation at cryogenic temperature This expansion in the plane of the 2D crystals is likely caused at least in part by the transition of covalent bonds to van der Waals contacts between the fragments produced by irradiation. It appears that in the competition between expansion due to covalent bond breakage, and potential cavity collapse and shrinkage following evaporation of volatile radiation products, it is the expansion that dominates.

### 4.3 Comparison with X-ray diffraction spot fading versus temperature

Similar effects to those described here occur during X-ray diffraction from 3D crystals of proteins. Meents et al. [28] showed that crystals of insulin or elastase, as judged by the reduction in spot fading rate, could tolerate a greater exposure by 23% to 18%, respectively, at 50K compared with 100K, but they did not find any further improvement between 50K and 5K. They also showed an increase in cell dimensions, which became greater as temperature was reduced down to 5K, similar to our observations for 2D crystals under electron irradiation. The exact amount of unit cell expansion in 3D crystals was found to be highly variable, even between different crystals of the same protein, in multiple X-ray crystallographic studies, ultimately rendering it not the best metric of radiation damage [49], [50]. Still, the magnitude of unit cell expansion observed in X-ray crystallography is similar to what we found in this work (0.1–1%). In X-ray diffraction, however, the increase in mosaicity is another important factor that can degrade the quality of diffraction data. The radiation-induced mosaicity of the frozen 3D crystals studied in [28] had a minimum at 50K, but increased again below 50K; this may be the reason why further improvements in $R_{\mathrm{free}}$ did not occur at temperatures below 50K. Mosaicity does not appear to be a factor in the electron diffraction from 2D crystals, possibly because 2D crystals consist of only a very thin single or double layer of proteins molecules, so that the 2D lattice is restrained by interactions with the surface of the supporting film. In any case, the issue of mosaicity will be of no relevance for single-particle cryoEM at liquid-helium temperature.

## 5 Conclusions and outlook

We have established that the structure of molecules of paraffin, bacteriorhodopsin and aquaporin in 2D crystals is preserved for longer in every case during irradiation at liquid-helium temperatures compared to liquid-nitrogen temperatures. None of these three specimens were embedded in frozen water or amorphous ice. The paraffin crystals consisted of dry straight-chain saturated hydrocarbons, whereas purple membranes and aquaporin 2D crystals were embedded in frozen, glassy glucose or trehalose. We cannot extrapolate directly from these observations to what will happen in frozen water. Further measurements of radiation damage are needed, especially for single particles embedded in amorphous ice. Nevertheless, we are optimistic that a similar improvement in radiation damage rate will carry over to specimens in vitrified water because the underlying mechanisms of radiation damage – ionisation, bond breaking, and diffusion of radiolysis products – are common to both. There is good agreement between the magnitude of the increase in B with electron irradiation that we observe (5–7 Å${}^{2}$/(e${}^{-}$/Å ^2^)) for spot-fading in 2D protein crystals at liquid-nitrogen temperature and the 5.0 ± 0.3 Å${}^{2}$/(e${}^{-}$/Å ^2^) slope of plots of B versus fluence in movement-free single-particle cryoEM dose-fractionated movies [51], also suggesting no fundamental differences in radiation damage mechanisms.

The impact of cooling from liquid-nitrogen to liquid-helium temperature on images, rather than diffraction patterns, of biological specimens is more complex. At lower temperatures some gases, such as oxygen, nitrogen and argon condense on all cold surfaces, including the specimen. Cryo-shielding, which is already required in cryomicroscopes to prevent condensation of water vapour onto the specimen at liquid-nitrogen temperature, can also protect the specimen from the condensation of any other gases at lower temperatures, but may need to be improved. Semi-conducting specimen support films, such as carbon, become more insulating as their temperature approaches 0K. Their electrostatic charging during irradiation with electrons can degrade image quality. Noble metal supports, such as gold, do not suffer from this problem. Their conductivity increases with lower temperature, meaning electrostatic charging of the specimen, prepared on all-gold supports, during imaging at liquid-helium temperature is not a significant concern. Motion of the specimen during irradiation, in the absence of stage drift, has mainly indirect effects (crystal expansion, tilt, bending) on the diffraction pattern which records only the intensity of the diffraction spots. In contrast, beam-induced specimen motion causes blurring of electron micrographs, and in the worst case, can cause a complete loss of all high-resolution features in an image. Previous investigations of the effects of radiation damage and beam-induced motion have proposed tradeoffs to maximise the quality of the processed images. Bammes et al. [52] evaluated images at four different temperatures between 4K and 100K and concluded that an intermediate temperature around 50K provided a good compromise between improved specimen lifetime and worse beam-induced motion. Pfeil-Gardiner et al. [8] carried out a full structure determination of apo-ferritin comparing data collected at liquid-helium and liquid-nitrogen temperatures. The resolution they obtained was virtually identical but with twice as much beam-induced motion at liquid helium. Their conclusion was that any reduction in the effect of radiation damage at the lower temperature was cancelled out by an increase in blurring due to specimen motion. Several methods have been proposed to reduce the movement in single-particle specimens for cryoEM. Some rely on improved all-gold or gold-molybdenum specimen supports [9], [53], and strengthening of the thin suspended ice film with the addition of a supporting graphene layer [23]. Another group of methods reduce specimen movement by annealing to reduce stresses in the vitrified water that are built up during the plunge freezing procedure. These include recrystallisation of the amorphous ice [54], or slower cooling of the specimen [55], including by vitrification in boiling nitrogen [56]. To date, only one method has demonstrated a reduction in the magnitude of beam-induced specimen motion to the theoretical minimum set by the pseudo-diffusion of water during irradiation at liquid nitrogen temperatures [57]. The method involves using small-hole all-gold specimen supports which constrain the ratio of hole diameter to ice thickness to less than 11:1 [51]. These specimen supports were designed to prevent the stresses in the amorphous ice from exceeding a critical value, and were demonstrated to reduce the specimen movement to less than 1 Å during a typical exposure.

There remains some uncertainty in the properties of amorphous water during electron irradiation at liquid-helium temperature, which can affect specimen movement at these lower temperatures. Wright et al. [58], following Heide & Zeitler [59], observed a change in the spacing from 3.7 to 3.3 Å of the broad electron diffraction peak from pure water after 2–3 e${}^{-}$/Å${}^{2}$ irradiation at liquid-helium temperature. They suggested that this observation implied a 30% increase in density due to a phase transition to a higher density form of amorphous ice, but other explanations are possible. Regardless of whether or not there is such a density change, the theory of critical stress developed in [51] can still be applied. We expect that ice movement can be eliminated with an appropriate specimen support aspect ratio, once the properties of the ice at liquid-helium temperature are known. Movement free imaging at liquid-helium temperatures may finally allow the capture of more information from electron micrographs acquired at these lower temperatures. The slower radiation damage at liquid-helium temperature would then allow more accurate extrapolation of structure factors to zero dose, before the onset of damage [51]. This potential improvement would be particularly useful for atomic-resolution structure determinations [60], [61], structure-based drug design [62], and structural studies of radiation-sensitive metalloenzymes [63]. Finally, the reduced radiation damage at liquid-helium temperature could improve the imaging of small particles in thick specimens [39], especially if the movement can be eliminated in these as well.

## Declaration of Competing Interest

The authors declare that they have no known competing financial interests or personal relationships that could have appeared to influence the work reported in this paper.

## Appendix A Supplementary data

MMC S1 .
