# Supplementary material for: On the reduction in the effects of radiation damage to two-dimensional crystals of organic and biological molecules at liquid-helium temperature
Source: Ultramicroscopy. 2022 Jul;237:None. doi: 10.1016/j.ultramic.2022.113512 (PMC9355890; doi:10.1016/j.ultramic.2022.113512)
Supplement: MMC S1 — . [file mmc1.pdf]

Appendix A: Supplementary Figures and Tables

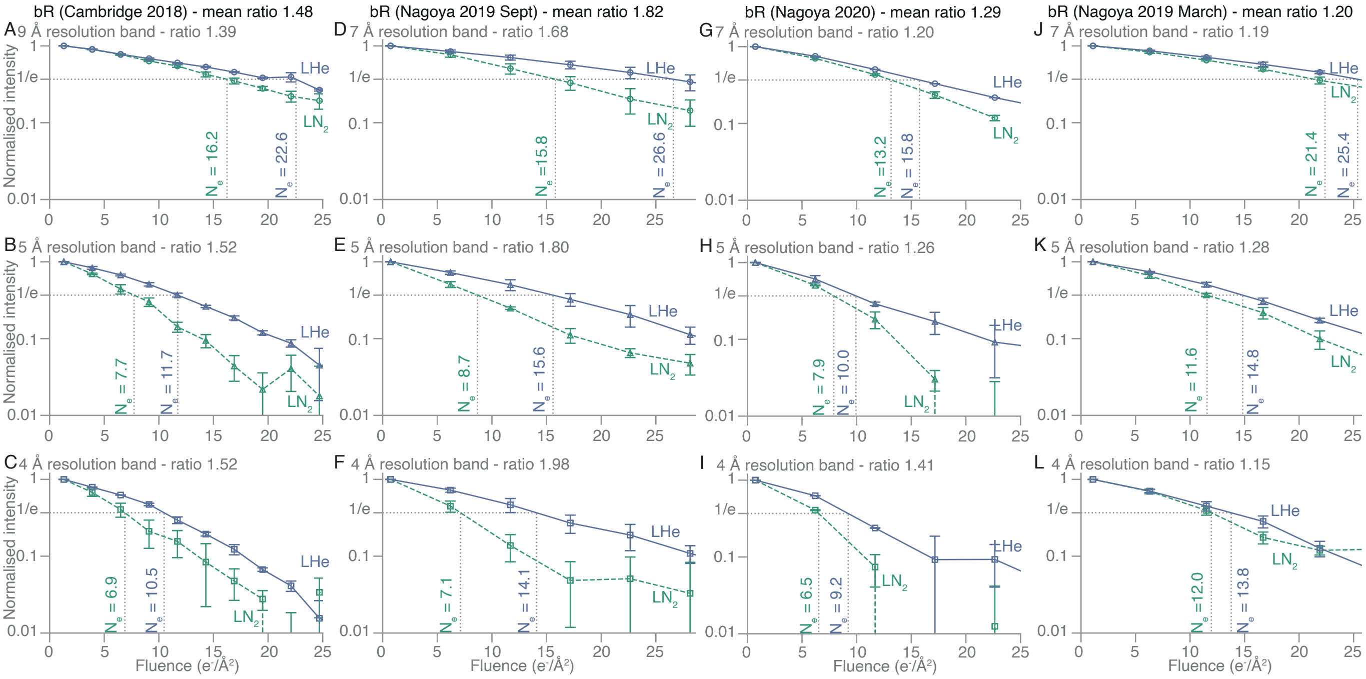

Figure A1: **Measurements of spot fading on purple membrane crystals at liquid-nitrogen (LN_2, *dashed green line*) and liquid-helium (LHe, *solid blue line*) temperature from four independent experiments.** Panels **(A-F)** show the same fading curves as in Figure 3, and panels **(G-L)** show the results of repeating the same experiment two more times on the microscope in Nagoya, with specimens prepared in Cambridge. For **(G-I)** $n = 2$ selected diffraction pattern series were averaged for each of the temperatures, and for **(J-L)** $n = 3$ for each temperature.

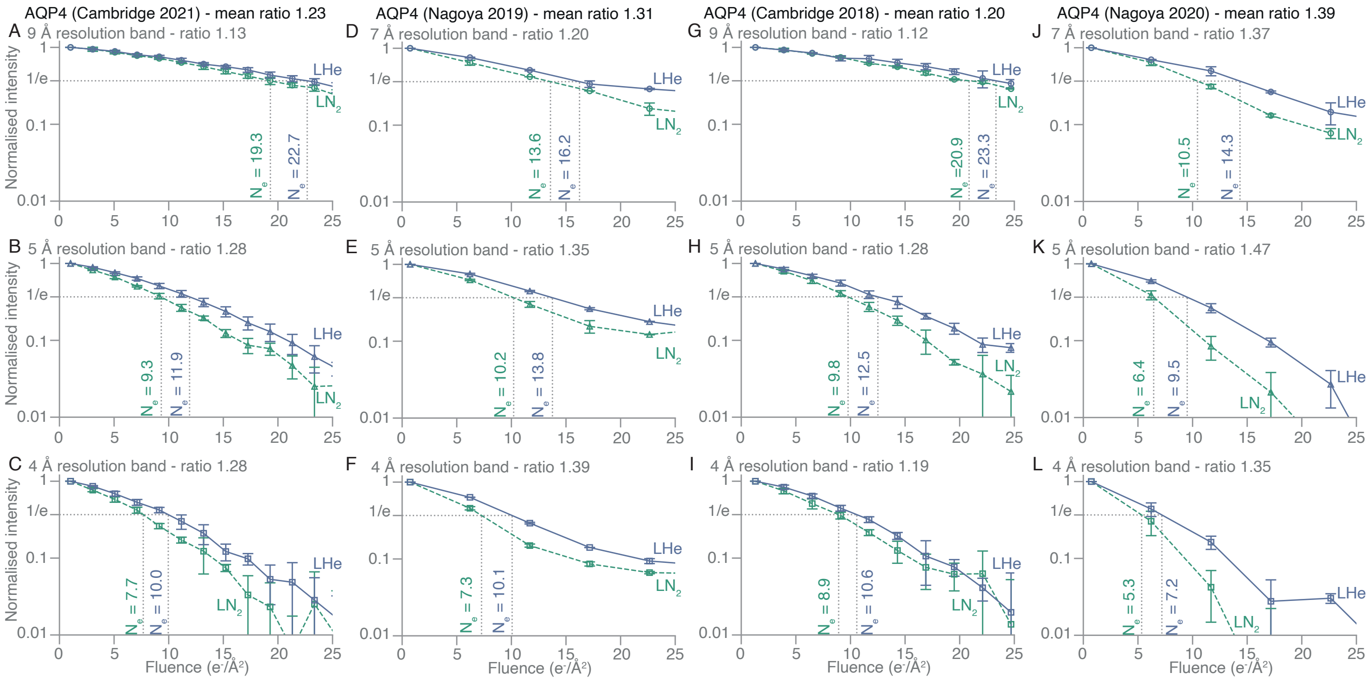

Figure A2: **Measurements of spot fading on aquaporin crystals at liquid-nitrogen (LN_2, *dashed green line*) and liquid-helium (LHe, *solid blue line*) temperature from four independent experiments.** Panels **(A-F)** show the same fading curves as in Figure 4, and panels **(G-L)** show the results of repeating the same experiments on the two different microscopes. For **(G-I)** $n = 3$ selected diffraction pattern series were averaged for each of the temperatures, and for **(J-L)** $n = 2$ for each temperature.

Publication	N_e Low Resolution (∞-7Å)			N_e High Resolution (7–4Å)		
	RT	LN_2	LHe	RT	LN_2	LHe
Siegel 1972, paraffin [15]	-	-	-	5 (3)	14 (9)	21 (14)
Fryer 1984, paraffin [5]	-	-	-	3 (1.7)	-	-
Brink+Chiu 1991, paraffin [16]	-	-	-	5 (2.5–4)	11 (10–14)	17 (7.5–14)
Hayward 1979, bR [11]	3 (1.8)	15 (9)	-	1.1 (0.7)	10 (6)	-
Stark 1996, bR [17]	1.7 (1.1)	6 (4)	11 (7)	-	1.7 (1.1)	3(1.9)
Unwin 1975, bR and catalase [18]	0.8 (0.5)	-	-	-	-	-
Fujiyoshi 1998, catalase [14]	-	-	-	(0.4)	(2)	(4–7)
Hattne 2019, proteinase K [19]	-	-	-	4.1 (3.6)	-	-
Hattne 2018, proteinase K [20]	-	-	-	-	(2–3)	-
Hattne 2018, hepta-peptide [20] (GSNQNNF)	-	-	-	-	(2–3)	-
This work, paraffin $C_{44}H_{90}$	-	-	-	-	14	22
This work, bR	-	16	25	-	7–8	11–15
This work, AQP4	-	16	19	-	8–9	10–13
Overall consensus N_e	2	16	20	1	8	12
ΔB-factor (Å^2/(e$^-$/Å^2))	60	8	6	50	6	4

Table A1: Summary of previous and present spot fading on variety of specimens. Radiation damage is measured by critical dose, N_e measured in e$^-$/Å^2, and rescaled to 300 keV electron energy. The scaling factors used were 1.55× for 100 keV, 1.14× for 200 keV, and 0.93× for 400 keV, according to [44]. The unscaled numbers from the cited publications are shown in parentheses. The N_e values at particular resolutions measured in e$^-$/Å^2 can be converted into ΔB-factors measured in Å^2/(e$^-$/Å^2) using Eqn. 2.
